# GAS2L1 Is a Potential Biomarker of Circulating Tumor Cells in Pancreatic Cancer

**DOI:** 10.3390/cancers12123774

**Published:** 2020-12-15

**Authors:** Lei Zhu, Ke-Jia Kan, Johanna L. Grün, Barbara Hissa, Cui Yang, Balázs Győrffy, Sonja Loges, Christoph Reißfelder, Sebastian Schölch

**Affiliations:** 1 Junior Clinical Cooperation Unit Translational Surgical Oncology, German Cancer Research Center (DKFZ), 69120 Heidelberg, Germany; lei.zhu@dkfz.de; 2 Department of Surgery, Universitäts Medizin Mannheim, Medical Faculty Mannheim, Heidelberg University, 68167 Mannheim, Germany; Kejia.Kan@medma.uni-heidelberg.de (K.-J.K.); johanna.gruen@umm.de (J.L.G.); bhissa@beilstein-institut.de (B.H.); cui.yang@umm.de (C.Y.); christoph.reissfelder@umm.de (C.R.); 3 Department of Bioinformatics and 2nd Department of Pediatrics, Semmelweis University, H-1094 Budapest, Hungary; gyorffy.balazs@med.semmelweis-univ.hu; 4 TTK Cancer Biomarker Research Group, Institute of Enzymology, H-1117 Budapest, Hungary; 5 Division of Personalized Medical Oncology (A420), German Cancer Research Center (DKFZ), 69120 Heidelberg, Germany; s.loges@dkfz.de; 6 Department of Personalized Oncology, University Hospital Mannheim, University of Heidelberg, 68167 Mannheim, Germany

**Keywords:** biomarkers, circulating tumor cells, single-cell RNA sequencing, liquid biopsy, pancreatic neoplasms, pancreatic ductal adenocarcinoma, mice, genetically engineered mouse model, computational biology

## Abstract

**Simple Summary:**

The analysis of circulating tumor cells (CTC) is a mainstay of liquid biopsy of solid malignancies. However, research to date has not yet determined a universal and specific marker for CTCs of pancreatic cancer. Genetically engineered mouse models (GEMMs) of pancreatic cancer, can mimic the human disease very closely. This study aimed to identify potential biomarkers for CTCs in a GEMM of pancreatic cancer and further validate markers in human samples. Therefore, we analyzed single-cell RNA sequencing data of murine pancreatic CTCs and performed advanced bioinformatic analyses. We demonstrated that the focal adhesion pathway is functionally enriched in pancreatic CTCs. In addition, we suggest Gas2l1/GAS2L1 as a potential surface marker of pancreatic CTCs. In combination with *Epcam/EPCAM, Gas2l1/GAS2L1* identify the majority of pancreatic CTCs. Furthermore, pancreatic cancer patients with overexpression of *GAS2L1* have an unfavorable prognostic outcome.

**Abstract:**

Pancreatic cancer is a malignant disease with high mortality and a dismal prognosis. Circulating tumor cell (CTC) detection and characterization have emerged as essential techniques for early detection, prognostication, and liquid biopsy in many solid malignancies. Unfortunately, due to the low EPCAM expression in pancreatic cancer CTCs, no specific marker is available to identify and isolate this rare cell population. This study analyzed single-cell RNA sequencing profiles of pancreatic CTCs from a genetically engineered mouse model (GEMM) and pancreatic cancer patients. Through dimensionality reduction analysis, murine pancreatic CTCs were grouped into three clusters with different biological functions. *CLIC4* and *GAS2L1* were shown to be overexpressed in pancreatic CTCs in comparison with peripheral blood mononuclear cells (PBMCs). Further analyses of PBMCs and RNA-sequencing datasets of enriched pancreatic CTCs were used to validate the overexpression of *GAS2L1* in pancreatic CTCs. A combinatorial approach using both *GAS2L1* and *EPCAM* expression leads to an increased detection rate of CTCs in PDAC in both GEMM and patient samples. *GAS2L1* is thus proposed as a novel biomarker of pancreatic cancer CTCs.

## 1. Introduction

Pancreatic ductal adenocarcinoma (PDAC) is the fourth most frequent cause of cancer-related death in western countries [1]. Fewer than one in five patients are eligible for potentially curative therapy since the tumor is unresectable in most cases at the time of diagnosis [2]. Even after resection with curative intent, most patients develop local or distant recurrence and ultimately succumb to the disease. Liquid biopsy, i.e., the detection and characterization of tumor-related molecules or intact tumor cells in a blood sample, is a tool that may enable clinicians to detect metastatic disease early on and thus avoid surgery in patients who may not benefit from resection due to occult metastatic disease.

Liquid biopsy is defined as investigating tumor biology in different blood-borne sources such as circulating tumor DNA (ctDNA) or circulating tumor cells (CTCs). CTCs are shed from the tumor, survive in circulation, and ultimately form distant metastases. In contrast to ctDNA, CTCs are viable tumor cells and, as such, they might represent a more accurate reflection of the current tumor biology compared to ctDNA also originating from dead cells. Furthermore, ex vivo culture of CTCs allows the downstream function experiments or even personalized translational research [3,4,5]. CTCs have been shown to have prognostic and predictive value in pancreatic cancer [6].

The extreme rarity of CTCs in many epithelial tumors has led to numerous CTC isolation technologies, which can detect CTCs based on physical properties or biologic markers [7]. Techniques that rely on physical properties to distinguish CTCs from blood cells by size, density, or even elasticity have unique advantages because they are unbiased and independent of cell surface markers, which might be present only in a subfraction CTCs depending on their biological state. However, to date, most techniques to detect CTC still depend on surface markers. The most common biomarker used to identify and isolate epithelial tumor-derived CTCs is the Epithelial Cell Adhesion Molecule (EPCAM). However, recent studies found that EPCAM-negative CTCs are also involved in the metastatic cascade [8,9,10], demonstrating the need for other markers to identify and isolate CTCs. Generally, CTCs are a heterogeneous cell population, and there is currently no uniform marker available which identifies all CTCs while excluding all other cells present in the blood [11,12,13,14]. As a way to overcome this problem, combinations of multiple markers have been used [15,16]; however, the currently available means to identify and characterize CTCs are still unsatisfactory.

This study aims to combine and analyze publicly available, single-cell RNAseq expression profiles of human and murine PDAC-derived CTCs to discover new biomarkers that could be potentially used to identify pancreatic cancer-derived CTCs.

## 2. Results

**Figure 1:**
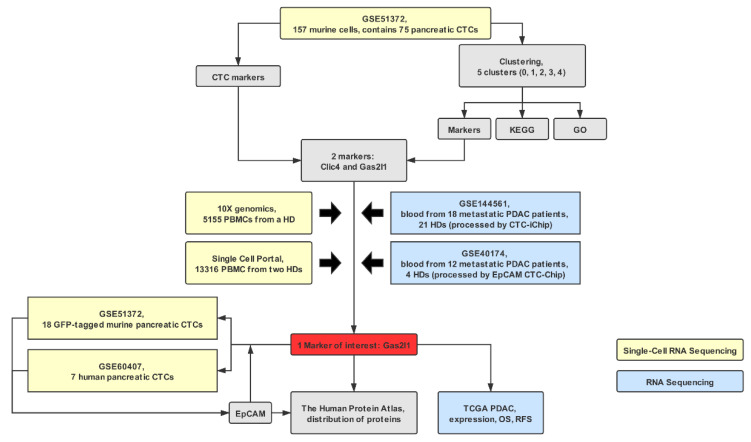
Study flow diagram. CTCs, circulating tumor cells; EPCAM, epithelial cell adhesion molecule; GO, gene ontology; HD, healthy donor; KEGG, Kyoto Encyclopedia of Genes and Genomes; PBMC, peripheral blood mononuclear cells; PDAC, pancreatic ductal adenocarcinoma; OS, overall survival; RFS, relapse-free survival.

The workflow of the analysis is depicted in Figure 1. 

### 2.1. Pancreatic CTC Heterogeneity Leads to Distinct Clustering

**Figure 2:**
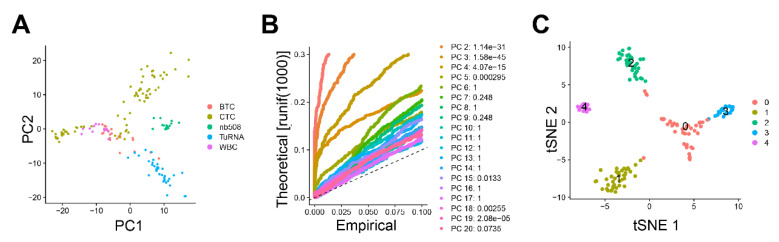
Clustering of murine cells after single-cell sequencing. There are 157 cells from the GSE51372 dataset that were taken into the analysis, including 75 circulating tumor cells (CTCs) from the genetically engineered mouse model (“CTC”), 34 primary tumor cells (“TuRNA”) (from which 10 or 100 pg RNA of diluted bulk RNA were taken), 20 EGFP-positive primary bulk tumor cells (“BTC”), 16 single tumor cells from NB508 cell line (“NB508”), and 12 WBCs from a control mouse (“WBC”). (**A**) Principal component analysis (PCA) of the cells. (**B**) JackStraw plot of the first 20 principal components (PCs). The distribution of p values for each PC with a uniform distribution (dashed line). The first five PCs show strong enrichment of features with low p values (solid curve above the dashed line). (**C**) t-Distributed Stochastic Neighbor Embedding (t-SNE) plot of 157 cells. All cells can be distributed into five clusters (cluster 0, 1, 2, 3, and 4). Cluster 0 consists of murine pancreatic CTCs, bulk tumor cells (BTCs), and white blood cells (WBCs). Cluster 1 only contains CTCs. Cluster 2 consists of diluted bulk RNA (TuRNA) and BTCs. Cluster 3 only contains CTCs and cluster 4 represents NB508 cells. BTCs, bulk tumor cells; CTCs, circulating tumor cells; PC, principal component; t-SNE, t-Distributed Stochastic Neighbor Embedding; TuRNA, RNA of the bulk tumor; WBC, white blood cells.

157 cells of the GSE51372 dataset were taken into the principal component analysis (PCA). Among these cells, there were 75 CTCs from the GEMM (henceforth referred to as CTC group), 34 primary tumor cell samples from the GEMM (TuRNA group) (from which 10 or 100 pg RNA of diluted bulk RNA were taken), 20 EGFP-positive primary tumor cells (bulk tumor cells (BTC) group), 16 single tumor cells from the NB508 cell line (NB508 group) and 12 white blood cells (WBCs) from a control mouse (WBC group). As shown in Supplementary Figure S1A,B, there are significant sequencing depth and gene detection differences among the different sample types, which could impair the reliability of the traditional sequencing data analysis. Upon PCA, CTCs formed two distinct clusters, one of which clustered with both BTC and, surprisingly, with WBC, while the second cluster was separate, indicating substantial heterogeneity among murine pancreatic CTCs (Figure 2A). The groups nb508 and TuRNA formed distinct clusters. 

As depicted in Figure 2B, there is a drop of significance levels after the first five primary components (PCs). These five PCs were taken into the subsequent t-Distributed Stochastic Neighbor Embedding (t-SNE) analysis. Here, all 157 samples were grouped into five distinct clusters (Figure 2C). Murine pancreatic CTCs were identified as three distinct clusters (clusters 0, 1, and 3), which is in line with the original study results [17]. While cluster 0 was a mixture of CTCs, BTCs, and WBCs, clusters 1 and 3 contained only CTCs and were therefore included in the functional enrichment analysis. 

**Figure 3:**
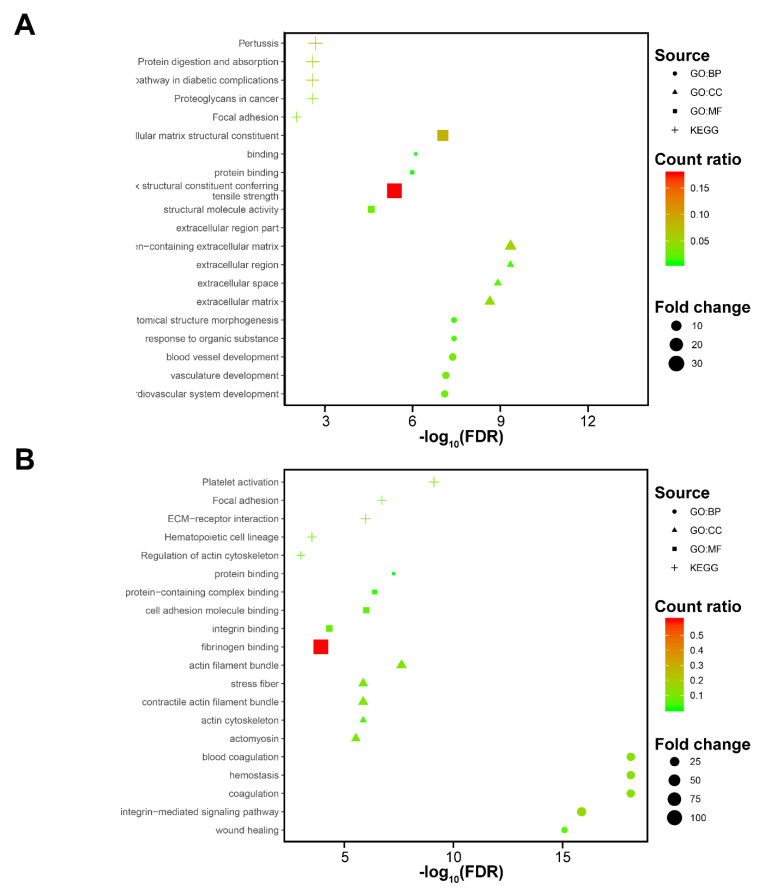
Functional enrichment analysis of cluster-specific markers. Functional enrichment analysis of genes in the clusters 1 (**A**) and 3 (**B**), top five enriched Kyoto Encyclopedia of Genes and Genomes (KEGG) pathways, Gene Ontology (GO) terms for biological processes (BP), cell components (CC) and molecular functions (MF) [fold enriched > 2 and false discovery rate (FDR) < 0.001]. BP, biological process; CC, cellular component; FDR, false discovery rate; KEGG, Kyoto Encyclopedia of Genes and Genomes; MF, molecular function; GO, gene ontology.

Cluster 1 comprised 39 of 75 CTCs (52.0%). Compared to the other four clusters, there are 139 genes upregulated and 15 transcripts with reduced expression in cluster 1. Further analysis (Figure 3A) showed that over-expressed genes were enriched in the proteoglycans in cancer pathway (KEGG: 05205; FDR = 2.79 × 10^−3^, fold change = 5.62). Consistently, the enriched gene ontology term proteoglycan metabolic process was significantly upregulated (GO: 0006029; FDR = 9.98 × 10^−3^, fold change = 9.28). 

Cluster 3 contains 26.7% (20/75) of pancreatic CTCs. Pathway analysis of cluster 3 signatures resulted in enrichment of the platelet activation pathway (KEGG: 04611; FDR = 7.74 × 10^−10^, fold changed = 13.88) (Figure 3B). Abundant terms associated with platelets were also enriched in the GO analysis, including platelet activation (GO: 0030168; FDR = 1.62 × 10^−13^, fold changed = 30.12), platelet aggregation (GO: 0070527; FDR = 7.78 × 10^−10^, fold changed = 35.94), regulation of platelet activation (GO: 0010543; FDR = 1.05 × 10^−3^, fold changed = 23.46 (Figure 3B). This strong platelet signal may be a result of platelets adhering to the CTCs.

Interestingly, the focal adhesion pathway was enriched significantly in both clusters ((KEGG: 04510; cluster 1: FDR = 9.82 × 10^−3^, fold changed = 4.97; cluster 3: FDR = 4.90 × 10^−7^, fold changed = 7.81). Further enriched GO annotations also support this result, such as actin cytoskeleton organization (GO: 0030036) and cell-matrix adhesion (GO: 0007160). 

### 2.2. Clic4 and Gas2l1 Are Overexpressed in Pancreatic CTCs

As the study’s primary aim was to identify biomarkers that can distinguish CTCs from blood cells, the expression levels of CTCs (group CTC) were compared to those of WBCs (group WBC). Interestingly, only nine genes were significantly over-expressed in CTCs, *Capns1*, *Csrp1*, *Rpl41*, *Bsg*, *Ppp2ca*, *Clic4*, *Gas2l1*, *Aldh2*, and *Fkbp8*. Two of them, *Clic4* and *Gas2l1*, were identified as markers of cluster 3. 

**Figure 4:**
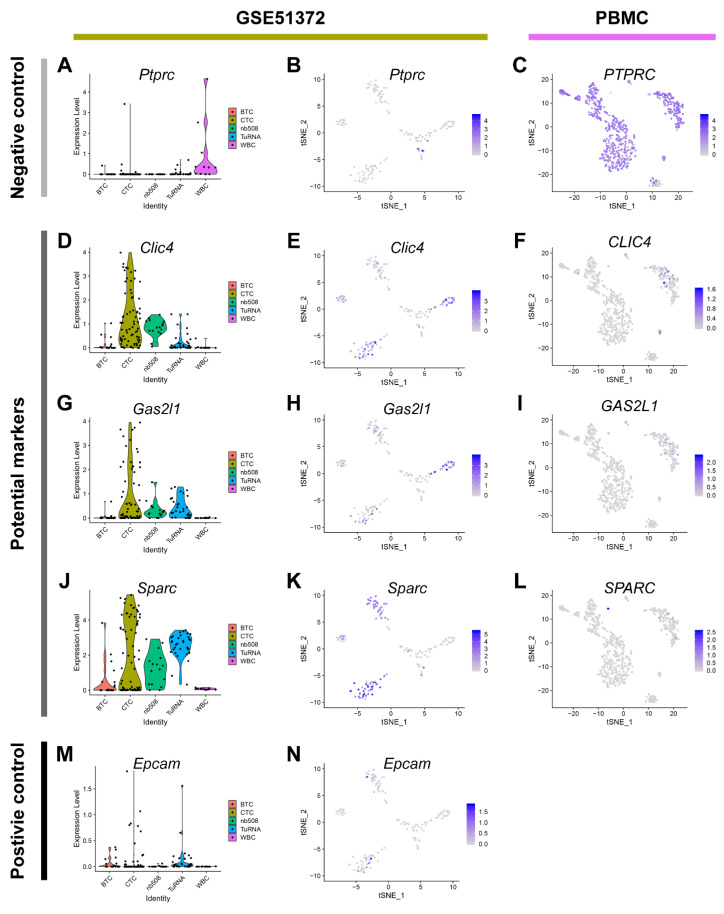
Expression of markers in circulating tumor cells (CTCs) and peripheral blood mononuclear cells (PBMCs). There are a violin plot and scatter plots for each marker to show the expression level in the CTC and PBMC datasets. *Ptprc/Cd45* (**A**–**C**) is a negative control, *Clic4* (**D**–**F**), *Gas2l1* (**G**–**I**) and *Sparc* (**J**–**L**) are potential CTC markers, *Epcam* (**M**,**N**) is used as the positive control. *Epcam* is not expressed in the PBMC dataset. BTCs, bulk tumor cells; CTCs, circulating tumor cells; t-SNE, t-Distributed Stochastic Neighbor Embedding; TuRNA, RNA of the bulk tumor; WBC, white blood cells.

The traditional leukocyte marker *Cd45 (Ptprc)* and the epithelial marker *Epcam* were used as negative and positive control markers, respectively. We also included *Sparc*, which was reported in the original study [17], into our analysis. While *Clic4*, *Gas2l1*, and *Sparc* were highly enriched in pancreatic CTCs (also surpassing the expression of *Epcam*), *Cd45* exhibited a low expression pattern in CTCs (Figure 4). To confirm the expression of candidate markers PBMCs from healthy humans, we use a single-cell RNA-seq dataset with 5155 PBMCs from 10x Genomics to perform the same analysis procedure. As expected, *CLIC4*, *GAS2L1*, and *SPARC* are rarely expressed in PBMC, while *CD45* (*PTPRC*) was ubiquitously expressed. This result was reproducible in another PBMC single-cell RNA sequencing dataset (Supplementary Figure S2).

### 2.3. Gas2l1 and Epcam Expression Identify Distinct CTCs Subpopulations and Synergize in CTC Identification

**Figure 5:**
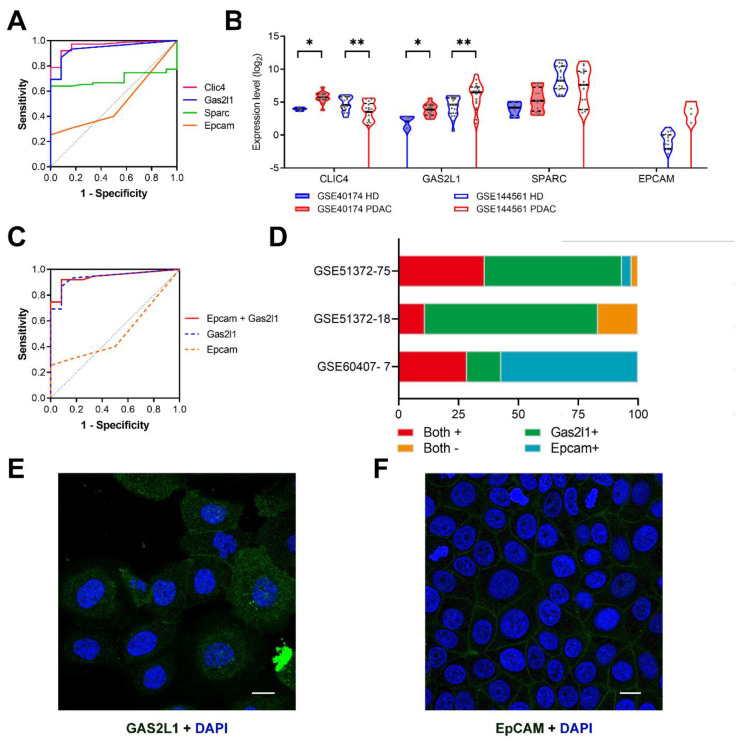
T *Gas2l1* is a potential marker for pancreatic circulating tumor cells (CTCs). (**A**) Receiver operator characteristic (ROC) analysis of markers in murine samples (GSE51372). (**B**) The violin plots reflect the expression level of *CLIC4*, *GAS2L1*, *SPARC*, and *EPCAM* in human blood samples (GSE40174 and GSE144561). *, *p* < 0.050; **, *p* < 0.001. (**C**) The ROC curve reflects the combination of *Gas2l1* and *Epcam* in murine samples (GSE51372). (**D**) The stacked bars show the *Gas2l1* and *Epcam* expression ratio in 75 murine pancreatic CTCs (GSE51372) and two validation datasets (18 GFP-Tagged murine CTCs from GSE51372 and 7 human pancreatic CTCs from GSE60407). The subcellular location of GAS2L1 (**E**) and EPCAM (**F**) in human cell lines (A-431 and MCF7, respectively); scale bar, 20 um. Blue, nucleus; Green, antibody. These two pictures were obtained from the Human Protein Atlas (https://www.proteinatlas.org/). AUC, area under curve; *EPCAM*, epithelial cell adhesion molecule; HD, healthy donor; PDAC, pancreatic ductal adenocarcinoma; ROC, receiver operator characteristic.

Further calculation of the AUC value and Mann–Whitney test of *Clic4*, *Gas2l1*, *Sparc* and *Epcam* were implemented. As depicted in Figure 5A, *Clic4* and *Gas2l1* exhibit significantly higher AUC values (AUC*_Clic4_* = 0.963, *p* < 0.001; AUC*_Gas2l1_* = 0.938, *p* < 0.001) than *Sparc* and *Epcam* (AUC*_Sparc_* = 0.695, *p* = 0.031; AUC*_Epcam_* = 0.514, *p* = 0.873). 

To validate whether the potential markers could work in enriched CTC samples, we also explored two other datasets, GSE40174 and GSE144561, which represent blood samples of PDAC patients processed by microfluidic CTC chips (EpCAM ^Hb^CTC-Chip [18] and CTC-iChip [19]). Remarkably, only *GAS2L1* is significantly overexpressed in the blood of metastatic PDAC patients in both datasets, while the differential expression of *CLIC4* and *SPARC* between the two groups is inconsistent (Figure 5B). As *GAS2L1* overexpression was found in these samples, we selected *Gas2l1* for the following analysis. 

The expression of *Gas2l1* was analyzed in 18 GFP-tagged murine pancreatic CTCs (GSE51372) and seven human pancreatic CTCs. While *Gas2l1* is expressed in most murine CTCs (83.3%, 15/18), three of seven human pancreatic CTCs (GSE60407) lack *GAS2L1* expression. This result indicates that similar to other known CTC markers, *GAS2L1* cannot identify all but only a subset of CTCs. In studies aiming to quantify all tumor-derived cells in the bloodstream, *GAS2L1* should therefore be combined with other markers such as *EPCAM* (Figure 5C). At least, *GAS2L1* is significantly overexpressed in CTC enriched cell population after the EpCAM ^Hb^CTC-Chip enrichment. In fact, the positivity of one or both markers identifies the majority of murine pancreatic CTCs (GSE51372; 73 of 75 CTCs (97.3%); 15 of 18 GFP-tagged CTCs (83.3%)) and all seven CTCs in the human pancreatic CTC dataset (GSE60407) (Figure 5D; Table 1). Interestingly, there is no statistically significant overlap in *Gas2l1*^+^ and *Epcam*^+^ murine pancreatic CTC populations (Figure 5D; Spearman r = −0.119, *p* = 0.310, Supplementary Figure S3), suggesting their complementary potential. The GAS2L1 protein is located both in the cytoplasm and the plasma membrane and the EPCAM protein is located in the plasma membrane (Figure 5E,F). Therefore, antibodies binding to GAS2L1 and EPCAM can be used to identify this CTC subpopulation without prior permeabilization of the cells. 

### 2.4. Intratumoral GAS2L1 Negatively Correlates with Recurrence-Free Survival

**Figure 6:**
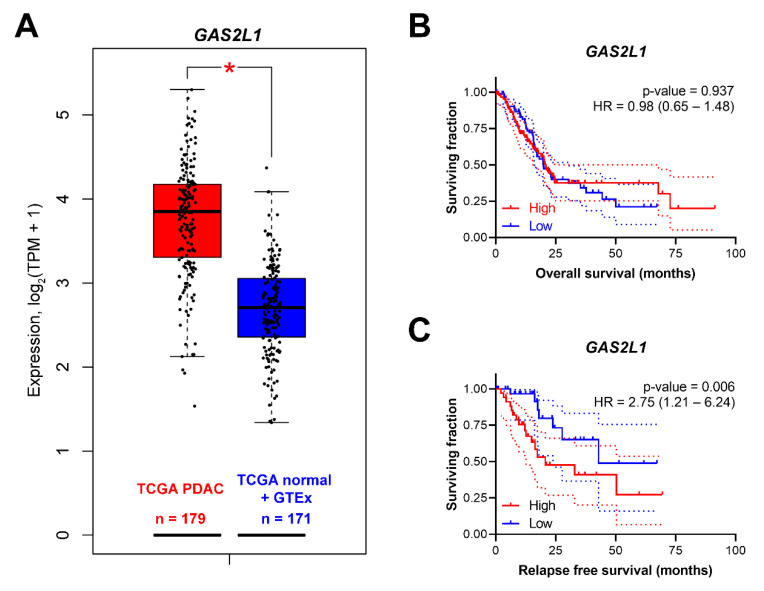
Prognostic value of *GAS2L1*. (**A**) *GAS2L1* expression in the TCGA pancreatic tumors with corresponding matched normal tissue and GTEx pancreatic tissue. *, *p* < 0.001. Overall survival (OS) (**B**) and relapse-free survival (RFS) (**C**) of *GAS2L1*. GTEx, The Genotype-Tissue Expression; HR, hazard ratio; PDAC, pancreatic ductal adenocarcinoma; TCGA, The Cancer Genome Atlas; TPM, transcripts per million.

To evaluate the expression of *GAS2L1* in pancreatic cancer and normal tissues, we utilized The Gene Expression Profiling Interactive Analysis 2 (GEPIA 2) (http://gepia2.cancer-pku.cn/#analysis) tool [20]. *GAS2L1* was significantly overexpressed (*p* < 0.001) in pancreatic adenocarcinoma as compared to matched normal tissue and the normal pancreatic data from the Broad Genotype-Tissue Expression (GTEx) portal (Figure 6A). We further investigated whether the expression levels of *GAS2L1* correlated with clinical prognosis (OS and RFS) in the pancreatic adenocarcinoma cohort of TCGA. Although OS was not influenced by *GAS2L1* [*p* = 0.937; HR = 0.98 (0.65–1.48)], patients with higher *GAS2L1* expression [*p* = 0.006; HR = 2.75 (1.21–6.24)] have significantly worse RFS (Figure 6B,C). These results point towards a prognostic value of *GAS2L1* in resected, non-metastatic pancreatic cancer.

## 3. Discussion

A growing body of evidence suggests that CTCs contribute to the development of metastases [5,21,22]. Besides, it is well established that CTCs can be found early in PDAC development [23,24,25,26]. In a genetically engineered mouse model (GEMM) of PDAC, pancreatic cells were detected in the bloodstream even before malignancy could be detected by histologic examination of the pancreas [27]. These observations encourage the hypothesis that CTC could be used as an early indicator of pancreatic malignancy. Furthermore, several research groups demonstrated that CTCs have prognostic relevance in pancreatic cancer [26,28,29,30]. 

It is still a technical challenge to distinguish CTCs from the surrounding blood components as a rare and heterogeneous population. Various technologies have been developed to isolate CTCs based on the fact that their physical properties (i.e., size, density, elasticity) differ slightly from those of leukocytes [7,31,32]. Alternatively, biologic differences such as protein expression on the cell surface can also be used to distinguish and isolate CTCs using fluorophore- or magnetic beat-coupled antibodies [33].

This study demonstrates that pancreatic CTCs are highly heterogeneous and can be divided into three distinct clusters, two of which are pure CTC clusters. Interestingly, both pure CTC clusters showed increased expression of the focal adhesion pathway and several relevant gene ontology terms, including actin cytoskeleton organization (GO: 0030036) and cell-matrix adhesion (GO: 0007160). 

The presented data also suggests that *Gas2l1* may be used as a potential identification marker for pancreatic CTCs. The role of *Gas2l1* (growth arrest specific 2 like 1) in cancer is largely unknown, and even more so in CTCs. Prior studies have noted that *Gas2l1* encodes a member of the growth arrest-specific 2 (GAS2) protein family, which guides microtubules towards focal adhesions through physical crosslinking of growing microtubules to actin stress fibers [34,35,36]. Only a few studies are investigating the role of GAS2 in oncogenesis and there is no consensus on whether GAS2 acts as a tumor suppressor or oncogene. GAS2 is upregulated in malignant glioma [37]. In colorectal cancer (CRC), fecal GAS2 was proposed as a non-invasive marker for early recurrence as it can be found in the feces of patients with recurrent CRC [38]. Besides, GAS2 expression is associated with proliferative activity in CRC [39]. In contrast, GAS2 seems to act as a tumor suppressor by inhibiting cell growth in hepatocellular carcinoma [40,41]. There is currently no data regarding the role of GAS2 in PDAC.

*GAS2L1* is required for centrosome and microtubule dynamics [34], which are required for cell polarization and migration [42,43]. Microtubules have a pivotal role in regulating cell protrusion and forming focal adhesions at the anterior migration margin [42,44]. As a result, microtubules are indispensable for CTC attachment to the capillary endothelium [45,46]. All of the above mechanisms are critically required for the successful completion of CTC seeding and, ultimately, the process of metastasis, suggesting a role of Gas2l1 in this process. 

*Gas2l1* is also reported to be expressed in platelets [46]. Multiple platelet pathways are overexpressed in cluster 3, most likely due to platelets adhering to the CTCs in this cluster. Therefore, it is reasonable to assume that the *Gas2l1* transcripts found in cluster 3 are at least partly derived from platelets. The CTC–platelet interaction may activate integrins to form a fibrin-based protective envelope for CTCs [46,47,48]. This may serve as a possible explanation for the link between worse RFS and overexpression of *GAS2L1* in human PDAC. However, this hypothesis is limited by the missing correlation between *GAS2L1* and OS in the clinical dataset, as well as the comparison between *GAS2L1* expression in tumor tissue and CTCs. In addition, only four of seven human pancreatic single CTCs expressed *GAS2L1*. This may reflect CTC heterogeneity or unknown confounding factors such as aspirin medication of the study participants. Generally, the available data from only seven human CTCs need to be put on a broader basis before drawing definitive conclusions.

The current literature recognizes the critical role of epithelial cell adhesion molecule (EPCAM) in identifying CTCs due to its absence in normal blood cells [49,50]. However, there is increasing concern that CTCs may at least partially lose epithelial traits, including EPCAM expression during epithelial–mesenchymal transition (EMT) [51,52,53,54,55]. For instance, only 40% (30/75) of the murine pancreatic CTCs in GSE51372 express *EPCAM.* Therefore, the usage of EPCAM as an identification marker in the majority of CTC studies leads to a severe bias toward epithelial CTCs. Several studies suggest that EPCAM-negative CTCs also have aggressive metastatic potential [56,57,58]. The addition of *GAS2L1* expression as a criterium to identify CTCs may increase the number of positively identified CTCs. As there is no significant overlap in the *Gas2l1*^+^ and *Epcam*^+^ CTC populations, GAS2L1 may be complementary to EPCAM as a selection marker in CTCs. This is supported by the fact that 7/7 human and 73/75 (97.3%) murine pancreatic CTCs were positive for either *GAS2L1* and/or *EPCAM*. Importantly, GAS2L1 protein can be found on the cell surface, therefore enabling the use of GAS2L1 antibodies for the identification and isolation of live CTCs without the need for prior permeabilization, which severely limits the use of subsequent RNA-based assays. This makes the combination of surface EPCAM/GAS2L1 a promising option for the identification of pancreatic CTCs. 

## 4. Materials and Methods 

### 4.1. Data Collection 

All datasets analyzed in this study are publicly available. Gene expression datasets and clinical information profiles for human PDAC were obtained from the Cancer Genome Atlas (TCGA) data portal (https://tcga-data.nci.nih.gov/tcga/) [59]. The RNAseq-based expression normalization fragments per kilobase of transcript per million mapped reads upper quartile (FPKM-UQ) normalization method was used. 

Two single-cell sequencing datasets of pancreatic CTCs were downloaded from the Gene Expression Omnibus (GEO) database (http://www.ncbi.nlm.nih.gov/geo/). GSE51372 contains CTCs from LSL-*Kras^G12D^*, LSL-*Trp53^flox/flox or +^*, *Pdx1*-*Cre* mice [17]. Another dataset (GSE60407) consists of seven expression profiles of CTCs from three PDAC patients [17]. Both datasets were generated on an AB 5500xl Genetic Analyzer on GPL15907 (*Mus musculus*) and GPL16288 (*Homo sapiens*) platforms, respectively. Another two RNA-seq datasets of enriched pancreatic CTC samples are also accessible through GEO Series accession number GSE40174 [60] and GES144561 [61].

Additionally, a single-cell gene expression dataset of 5155 peripheral blood mononuclear cells (PBMCs) of a healthy donor was obtained from 10X genomics (https://support.10xgenomics.com/single-cell-gene-expression/datasets/3.0.2/5k_pbmc_v3_nextgem). The dataset was established on the Illumina NovaSeq platform, and sample demultiplexed, barcode processed, and single-cell 3’ gene counted by Cell Ranger 3.0.2. Single-cell expression profiles of PBMCs from healthy donors were downloaded from the Broad Single Cell Portal (BETA) (https://singlecell.broadinstitute.org/single_cell) and used for validation.

### 4.2. Data Quality Check, Pre-Processing, and Clustering

*Seurat,* a specific R toolkit for quality control (QC) and exploration of single-cell transcriptomic data, was employed according to the instructions provided by the Satija lab (https://satijalab.org/) [62]. 

After removing cells with fewer than 200 unique genes per cell from the dataset, we performed a global-scaling normalization, multiplied all remaining gene expression by 10,000, followed by log transformation. After normalization, the top 3000 highly variable genes were extracted from each dataset based on mean variance. 

Both linear (Principal Component Analysis (PCA)) and non-linear dimensional reduction (t-Distributed Stochastic Neighbor Embedding (t-SNE)) were performed after the scaling (linear transformation). The JackStrawPlot function was employed to calculate the top principal components of the dataset. Principal components (PCs) with low p-values after random permutation and PCA recalculation were identified as significant and submitted to the following clustering using the FindClusters function to iteratively group cells together based on modularity optimization techniques.

### 4.3. Statistical Analysis and Visualization

The cluster of interest was compared to all other clusters to identify the markers of the target clusters. *p* < 0.050 and │log(Fold Change, FC)│> 1 were chosen as cutoffs to define significant markers.

The functional enrichment analysis, which consists of the pathway analysis of the Kyoto Encyclopedia of Genes and Genomes (KEGG) [63] and the functional interpretations of Gene Ontology (GO) [64], was completed by the g:Profiler (https://biit.cs.ut.ee/gprofiler/gost) [65]. All three aspects—biological processes (BP), cellular components (CC), and molecular functions (MF)—were included in the GO analysis. Fold enrichment > 2.0 and Benjamini–Hochberg false discovery rate (FDR) < 0.001 were defined as statistically significant.

The prognostic value of GAS2L1 was explored in the TCGA pancreatic cancer cohort. Overall survival (OS) and relapse-free survival (RFS) curves were displayed with p-values (log-rank test) and 95% confidence intervals (CI) of hazard ratios (HR). The Kaplan–Meier plots were generated by GraphPad Prism 8 to visualize the differences and comparisons with *p* < 0.050 were considered statistically significant.

The expression in different groups was compared using the Receiver Operating Characteristic (ROC) and Mann–Whitney tests in the R statistical environment using Bioconductor libraries (www.bioconductor.org). Markers with an area under the curve (AUC) value of the ROC check > 0.70 and *p* < 0.050 were considered statistically significant. GraphPad Prism 8 was used to plot the figures.

## 5. Conclusions

In summary, the here presented data suggests Gas2l1/GAS2L1 as a potential biomarker for pancreatic CTCs. The combination of EPCAM/GAS2L1 surface protein stainings may increase the detection rate and comprehensiveness of CTC studies in pancreatic cancer without limiting the availability of the isolated CTCs for downstream analyses. As the available single-cell RNA-seq datasets of pancreatic CTCs are very limited in sample size, we utilized several complementary datasets to validate our findings. However, further experiments including murine and human studies, are necessary to evaluate the viability of the proposed GAS2L1 and EPCAM combination strategy in identifying pancreatic CTCs.

## Figures and Tables

**Table 1:** Expression of *GAS2L1* in CTC samples.

Dataset–Sample Size	*Gas2l1* ^+ 1^	*Epcam* ^+^	*Gas2l1*^+^ and/or *Epcam*^+^	Total
GSE51372-75	70 (93.3%)	30 (40.0%)	73 (97.3%)	75
GSE51372-18	15 (83.3%)	2 (11.1%)	15 (83.3%)	18
GSE60407-7	3 (42.9%)	6 (85.7%)	7 (100.0%)	7

^1^ +, positive expression.

## Supplementary Materials

The following are available online at https://www.mdpi.com/2072-6694/12/12/3774/s1, Figure S1: Visualization of the quality check metrics of 157 single-cell sequencing profiles; Figure S2: Expression of markers in peripheral blood mononuclear cells (PBMCs) from two healthy donors; Figure S3: *Gas2l1* and *Epcam* expression do not correlate in murine pancreatic CTCs.
