# Supplementary material for: GAS2L1 Is a Potential Biomarker of Circulating Tumor Cells in Pancreatic Cancer
Source: Cancers (Basel). 2020 Dec 15;12(12):3774. doi: 10.3390/cancers12123774 (PMC7765300; doi:10.3390/cancers12123774)
Supplement: Supplementary file 1 [file cancers-12-03774-s001.pdf]

SUPPLEMENTARY MATERIALS

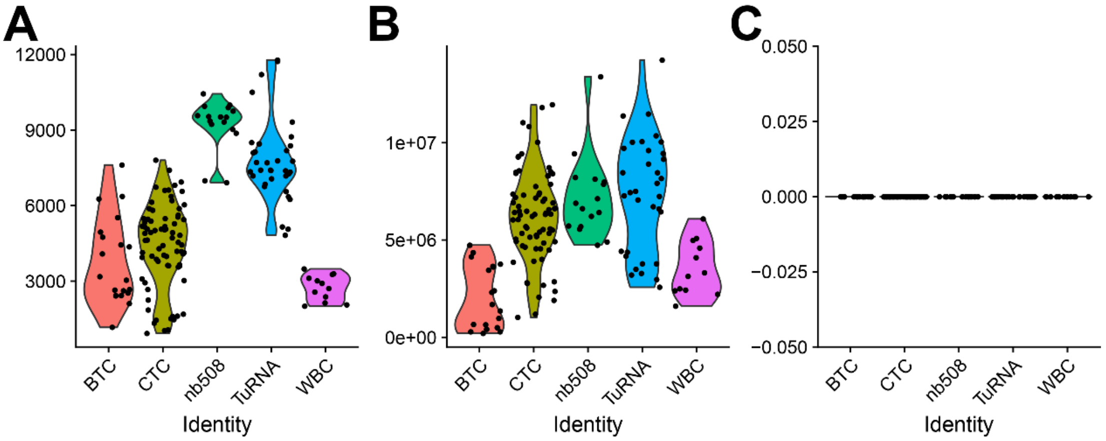

Supplementary Figure S1. Visualization of the quality check metrics of 157 single-cell sequencing profiles. (**A**) The number of unique genes detected in each cell. (**B**) The total number of molecules detected within a cell. (**C**) Percentage of reads that map to the mitochondrial genome.

BTCs, bulk tumor cells; CTCs, circulating tumor cells; t-SNE, t-Distributed Stochastic Neighbor Embedding; TuRNA, RNA of the bulk tumor; WBC, white blood cells.

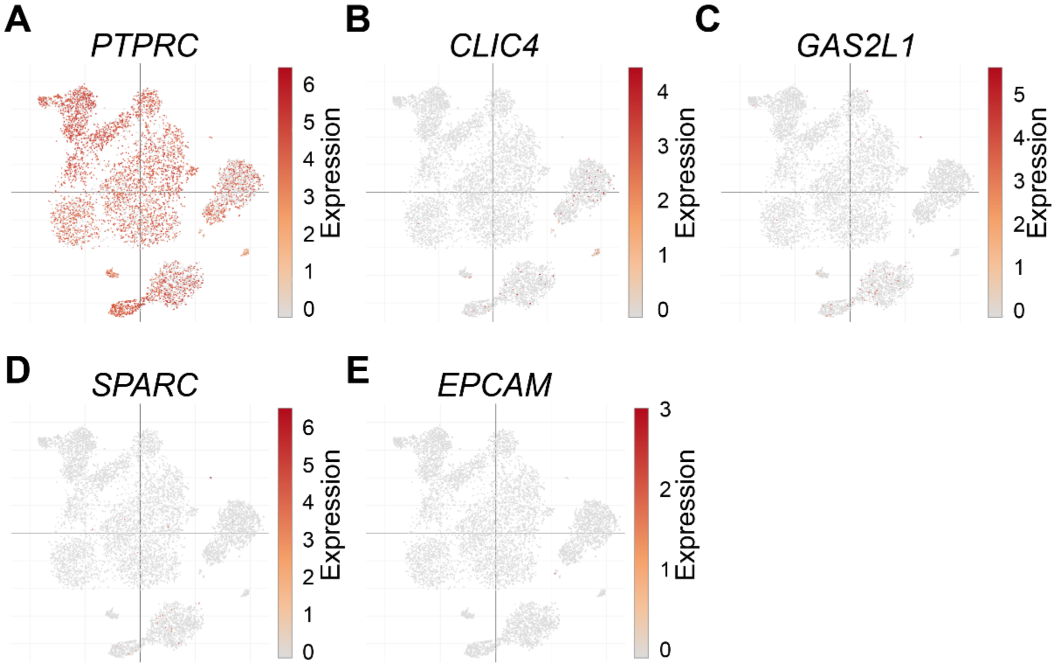

Supplementary Figure S2. Expression of markers in peripheral blood mononuclear cells (PBMCs) from 2 healthy donors. The dataset which contains 13,316 PBMCs is publicly available on the Single-Cell Portal (BETA) (https://singlecell.broadinstitute.org/single_cell). The t-distributed Stochastic Neighbor Embedding (t-SNE) coordinates show the expression of *PTPRC* (**A**), *CLIC4* (**B**), *GAS2L1* (**C**), *SPARC* (**D**), and *EPCAM* (**E**).

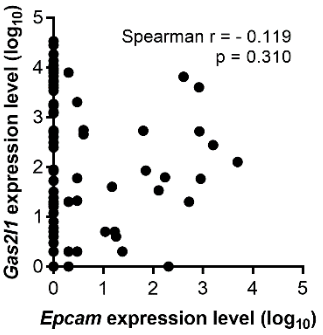

Supplementary Figure S3. *Gas2l1 and Epcam expression do not correlate* in murine pancreatic CTCs. Expression level = log_{10}(read counts + 1).
